# Implementing Supported Employment. Lessons from the *Making IPS Work Project*

**DOI:** 10.3390/ijerph15071545

**Published:** 2018-07-21

**Authors:** Jan Hutchinson, David Gilbert, Rachel Papworth, Jed Boardman

**Affiliations:** 1 Centre for Mental Health, Unit 2D21, Technopark, South Bank University, 90 London Road, London SE1 6LN, UK; jedboard@atlas.co.uk; 2 InHealth Associates, London EN4 8QA, UK; davidgilbert43@yahoo.co.uk; 3 Papworth Research & Consultancy Ltd., Brighton BN2 3PH, UK; rachel@papworth.info; 4 Institute of Psychiatry, Psychology and Neuroscience, King’s College, London SE5 8AF, UK

**Keywords:** supported employment, individual placement and support, mental health

## Abstract

Individual Placement and Support (IPS) is an internationally accepted and effective form of supported employment for people with severe mental health conditions. Despite its strong evidence base, the implementation of IPS has been slow and inconsistent. In England, a demonstration project, *Making IPS Work*, was developed to offer support for the implementation of IPS in six local sites National Health Service Mental Health trusts. The project aimed to: Establish Individual Placement and Support services within clinical teams; develop high fidelity practice and leave a sustainable IPS service beyond the project. The number of people gaining open employment in each site was monitored. Fidelity checks were carried out at three sites by independent assessors. Stakeholders were interviewed over the 18-month lifetime of the implementation period to examine the experience of developing the services in the six sites. A total of 421 jobs were found for people with mental health conditions over 18 months with a large variation between the highest and lowest performing sites. The sites assessed for fidelity all attained the threshold for a ‘Good Fidelity’ service. The new services were readily accepted by mental health service users, clinical staff and managers across the trust sites. Maintaining the funding for the Individual Placement and Support services beyond the project period proved to be problematic for many sites. Placing the services within a broader strategy of improving psychosocial services and bringing together decision making at the corporate, commissioning and clinical management level were helpful in achieving success. The growth and maintenance of these services is difficult to achieve whilst the current cost pressures on the NHS continue.

## 1. Introduction

**Figure 1:**
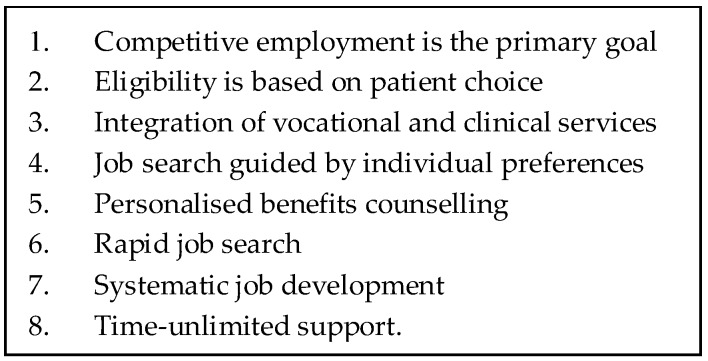
The principles of IPS Supported Employment.

One approach to vocational rehabilitation for people with severe mental health conditions, supported employment, has the primary aim of helping people get into a job that pays at least the minimum wage and is available on the open market (i.e., jobs not specially set aside for people experiencing mental ill-health). The most widely-researched supported employment approach is Individual Placement and Support (IPS) [1]. Based on eight principles (Figure 1), this person-centred approach focuses on finding job opportunities for people, working with potential employers to create suitable vacancies and providing ongoing support to the employee and employer. There is a considerable evidence base for the efficacy of IPS [1]. A recent systematic review of 17 international randomised trials provided consistent positive evidence for the success of IPS in helping people achieve open employment showing that it is 2.4 times as effective as other approaches to vocational rehabilitation [2]. IPS is cost-effective, has lower drop-out rates and generates positive outcomes across several domains (into work quicker, work more hours per week, longer job tenure). It gives good personal outcomes, fewer hospital admissions, and quicker recovery when compared with other approaches to vocational rehabilitation [1]. Working in line with the eight principles (‘fidelity to the model’) results in better employment outcomes [1,3]. Implementation fidelity, the degree to which an intervention or programme is delivered as intended [4], is thus important in determining the quality of and outcomes of IPS programmes and a lack of fidelity hampers the success of IPS programmes and their dissemination [5]. The use of IPS is supported internationally and IPS services have been set up in many countries (e.g., [1,3,6,7,8,9]). In the United Kingdom, IPS is embedded in government policy: The UK government plans to increase the employment opportunities for people with mental health problems and the *Five Year Forward View for Mental Health*, which sets out the plans for mental health services, aims to double the number of people accessing IPS services [10].

Despite the wealth of evidence for the efficacy of IPS and international support for its use, the implementation of IPS services has been slow and patchy. A national audit carried out in England in 2017 found that only 21 teams were delivering IPS to a total of around 9000 individuals at any one time [11]. Some of the key barriers to the implementation of IPS identified in the UK context include attitudinal barriers relating to the beliefs of clinicians, employers and others; contextual factors relating to the structure of the labour market and welfare systems; and organisational factors within mental health services [12].

Schemes have been designed to supply technical support to services and staff to aid the effective implementation of many psychosocial rehabilitation programmes including IPS (e.g., [13,14,15]). In the USA, the National Evidence-Based Practices Project [16] and the Mental Health Treatment study [17] both demonstrated that it is possible to implement local IPS schemes with high fidelity through the use of local support to champion good practice, feedback of the findings of fidelity reviews and encouragement of integration of vocational workers in clinical teams. The development of the learning collaborative in the Johnson and Johnson—Dartford County Mental Health Programme created state trainers to support networks of hospitals or clinical teams to share good practice, and set benchmarks and targets to attain and measure progress to monitor success. This programme has grown from demonstration sites in three US states into an IPS Learning Network covering 20 states and three European countries [18]. In 2014, a 2-year follow-up of 129 sites that participated in the learning network found that 96% of these sites sustained their activities, maintaining quality improvement, expanding funding and increasing employment rates and fidelity to the IPS model [19].

In England, the Centre for Mental Health has led the work to increase the coverage and quality of the implementation of IPS within health services. Centre for Mental Health has run IPS training courses and worked with partners to organise complementary training in motivational interviewing and employer engagement as well as providing IPS consultancy and coordinated implementation support. They developed a version of the Dartford programme: the *Centres of Excellence Programme* (see https://www.centreformentalhealth.org.uk/ips-centres-of-excellence) and piloted a regional trainer [15]. These approaches attempted to facilitate cultural change within an organisation, for example by encouraging an acceptance of employment as an integral part of a person’s recovery. They also use IPS fidelity reviews to determine how well the principles of IPS are applied in practice and feedback of these results to promote improvement in working practices. Since its inception, the Centres of Excellence Network has grown from eight schemes to 19 over the last decade. It has encouraged the development of shared resources, such as job descriptions, data monitoring tools and an anglicised version of the fidelity review tool. The regional trainer pilot achieved a significant increase in the number of job outcomes achieved by the integrated service in one English county (Sussex); this success was credited to the efforts of the particular regional trainer in increasing appreciation of IPS among referring clinicians, improving the skills of IPS employment specialists through training, and focusing efforts on rapid job search and on reaching job outcome targets [15].

The Centre for Mental Health went on to replicate the Regional Trainer pilot on a larger scale. This paper reports on the evaluation of that programme, *Making IPS Work*, designed to offer support for the implementation of IPS in six sites in England. The main aims of the project were to: Set up and embed the IPS employment service within clinical teams; achieve high fidelity practice over 18 months; and leave a sustainable IPS service operational beyond the grant-funded period.

This paper reports on an evaluation of *Making IPS Work*. The aims of the evaluation were to examine whether the implementation was addressing the particular circumstances encountered in each of the sites, and to explore features about the local implementation of IPS that would help others in other parts of the country to successfully implement IPS services.

## 2. Materials and Methods

### 2.1. Making IPS Work Project

The objective of the development project was to help local NHS provider organisations (NHS Trusts) to implement IPS services in their local area, by focusing on new exemplars of best practice. This was done in response to previous requests from NHS trusts to help them develop new IPS services in areas of England which had no Centres of Excellence at the time. Six sites: Bradford; Lincolnshire; Northamptonshire; Bedfordshire; Berkshire and Wiltshire, volunteered to be supported (see Table 1). The project was funded by the Department of Health in 2014. Work began in three sites by the end of 2014 and in the second wave of sites during 2015.

The project plan for this grant-funded piece of work was based on the fact that IPS is described by 25 items on the fidelity scale. These items cover some aspects of the set-up and delivery of the service from the support of the NHS executive directors for IPS through integration of supported employment with mental health treatment to the role that the employment specialists undertake. The use of the IPS manual and the training given by Centre for Mental Health staff to the vocational specialists helped to develop an approach based on the model fidelity (for example, how vocational specialists can build a supportive relationship with each service user, how they can support people into jobs of their preference, how they may approach employers to make a good match).

The funding was used to create two full-time posts for each site, both employed by Centre for Mental Health: An implementation manager (also known as an *IPS Regional Trainer*) and a team leader for the new IPS employment specialists. The team leader would supervise IPS practice and co-ordinate the IPS provision, overseeing staff who could be employed by a number of different local organisations. The title of this post varied across sites: IPS Team Supervisor, Team Leader, Service Manager or IPS Co-ordinator. In addition, a small amount of funding was offered to the NHS trusts to recognise the costs of hosting Centre for Mental Health staff. No funding was offered to pay the salaries of the IPS team members and participating NHS Trusts would be expected to provide, or second from other organisations, 4–6 employment specialists and to find the means to continue funding the IPS service beyond the lifetime of the grant (see Table 1).

In each site, the employment specialists were expected to work with a minimum of 120 people referred for supported employment and to successfully assist a minimum of 60 into paid work over an 18-month period. These job outcome targets for the number of job outcomes to be achieved were based on the experience of the Centre for Mental Health staff who have supported organisations to run IPS over at least the last 10 years. On average, new IPS services have been able to support 10–12 people per employment specialist into work in the first year and around 15–18 people per staff member in the second year. Allowing for the fact that new staff might not come into their post quickly in the first year it was felt that 60 as an overall target per site would be challenging, but not impossible—it was achieved in five out of six sites.

Job descriptions required staff to run an IPS service with high fidelity to the model and for the implementation manager to find alternative sources of funding and/or staff resources which would enable the IPS project to flourish beyond the timescale of the Department of Health grant.

Each site had a senior member of NHS staff who voluntarily took responsibility for the implementation of IPS within their trust. These managers took part in the local recruitment for the two Centre for Mental Health posts. The details of the appointments to the grant-funded posts varied across sites. For example, in the three first-wave sites five Centre for Mental Health staff were appointed and provided with honorary contracts by the NHS organisation, thus allowing them access to all trust information and data. They were also provided with laptops and phones by the trust sites. In one site, the candidate successful in obtaining the team leader role was already a member of staff of the organisation and was employed locally. Only one of the six staff had any prior experience of IPS, the remainder were recruited on the basis of their theoretical knowledge of IPS and relevant transferable skills and knowledge. Three of the six staff for the stage 2 sites staff were existing trust employees who moved across to the IPS project, joined by three new appointments.

In each site, the implementation managers’ roles included embedding IPS practice; facilitating support for the employment service at a strategic level in the local NHS trust; and influencing local commissioning and provision to ensure a legacy of IPS once the grant funded period was over. The IPS team supervisor’s role was to mentor, train and oversee high fidelity IPS practice and to support each team to achieve their job outcomes.

All the employment specialists, team leaders and implementation managers were provided with a 2-day training course in IPS. They also found it helpful to arrange group telephone calls among those employed in similar roles in the different sites. Supervision and management for the job role was provided by a Centre for Mental Health director for the implementation managers; by the implementation managers for the team coordinators and by the team coordinators for the IPS employment specialists.

In May 2017, all six sites had completed the grant-funded implementation project (18 months per site).

### 2.2. Recording of Quantitative Data

Each site collected data on the number of individuals referred and taken on by the IPS services and who had obtained suitable employment in the open market. As a means of ensuring that the numbers and characteristics of service users would be readily available, a spreadsheet was used to record the identity numbers of service users and their progress. The numbers of applications made by individuals and the number of interviews/meetings with employers was also recorded. This information was also useful for supervisors in monitoring whether activity to achieve a job placement was taking place. At the end of each month the implementation manager was required to send the spreadsheet to the Centre for Mental Health Project Manager and an up-to-date record was kept of the cumulative job outcomes, which produced an overall graph of performance against target.

### 2.3. Sources of Qualitative Evidence

Interviews and focus groups were carried out with staff, service users and local stakeholders across the six sites (Table 2). The data was collected using a semi-structured interview, undertaken by two interviewers separately (D.G. and R.P.) dividing the sites between them. The data was analysed into themes manually, separately by the reviewers, they then came together to discuss the emerging themes which suggested themselves as the data from all six sites was compared and carefully re-read.

Three-hundred individuals (235 members of staff and 65 service users) took part across the sites and included: employment specialists; team leaders; implementation managers; community mental health team (CMHT) staff; CMHT managers and clinical leaders; senior managers and leaders within the host organisation; service user engagement leads; external stakeholders (commissioners, local authority representatives, voluntary sector organisations); IPS clients. The data was collected during three site visits, approximately 7 to 8 months apart, beginning at commencement of the projects in each site, which enabled observation of progress across the sites.

Topic guides were used to structure the interviews and focus groups. The topic guides were developed by considering the over-arching theory of change, i.e., what would need to be put in place during the implementation to achieve a quality, functioning and sustainable service. Questions for stakeholders which drew out their views on aspects of that theory were developed by the evaluators. The main focus was to examine the degree of success of the implementation project. The purpose of the qualitative data collection was to identify elements of successful implementation and barriers to progress. This paper reports on these under four themes: the benefits of IPS, implementation challenges and opportunities, organisational factors, and sustainability.

## 3. Results

### 3.1. Employment Outcomes

The referrals seen in each site and the jobs achieved are shown in Table 3.

**Figure 2:**
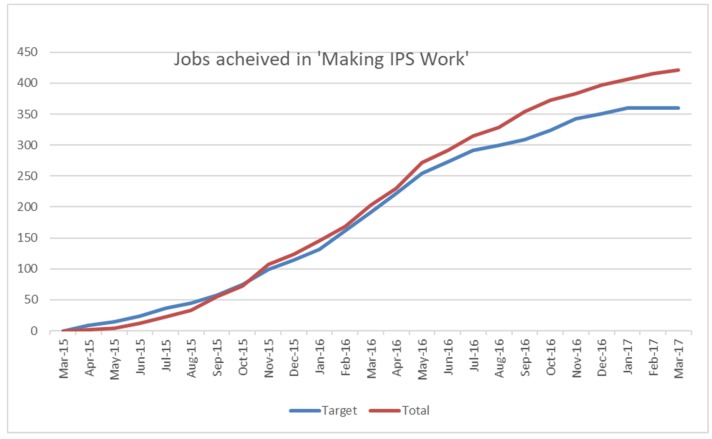
Jobs achieved in all sites.

The overall success in achieving the employment targets is illustrated in Figure 2, which shows the target and actual employment outcomes across the six sites. The total jobs achieved exceeded the target of 360. All sites had achieved their target of 60 job starts, with the exception of one site, Northamptonshire, which began taking referrals at a later stage of its implementation by comparison with other sites and achieved its target by the end of May 2017. The total number of jobs achieved by the end of the project in March 2017 was 421.

**Figure 3:**
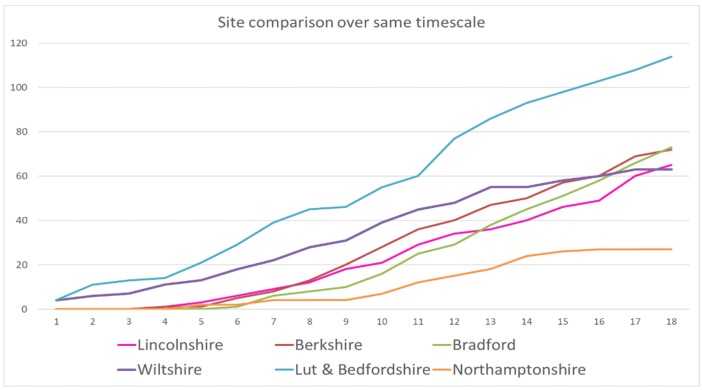
Jobs achieved: Comparison of sites over 18-month period.

There was a large variation between the highest and lowest performing sites (Figure 3). Bedfordshire and Luton which had the highest number of staff and some existing experience of delivering IPS achieved 114 job starts. Northamptonshire, which experienced some staff performance challenges, achieved 27 jobs starts. In the period following the grant-funded project, a higher rate of job outcomes was achieved, perhaps because staff performance issues had been resolved. The achievements of the projects during the grant-funded 18 months period was normally distributed: with higher achievement by Bedfordshire and Luton, lower by Northamptonshire and the other four sites bunched in the middle, just above the target of 60 job outcomes.

### 3.2. Fidelity Measures

Three sites were assessed for fidelity by reviewers independent of both the service and Centre for Mental Health. They all scored over 100 out of 125, which is the threshold for a ‘Good Fidelity’ service. The other three sites chose not to be reviewed as they felt, at just 18 months from start-up, and with the sustainability of posts remaining uncertain, that it was too soon to expect a high-fidelity score.

### 3.3. Qualitative Outcomes

#### 3.3.1. The Distinctiveness of IPS

In each area, the people interviewed for the evaluation were overwhelmingly positive about IPS: in terms of its effectiveness in supporting people to get work; its wider benefits to people’s confidence, skills, wellbeing and daily life; and the knock-on effects on staff morale and confidence within teams.

“*Having* [an Employment Specialist] *in place has helped; we knew it was going to make a massive difference… helps ease burden, freed a lot of time. Eight out of ten clients who try the traditional job centre approach fail. The difference is in the practical help in IPS; clients would struggle to go to find jobs on their own; the help they get is great, practical, CVs etc had one client who calmed down a lot under* [Employment Specialists] *support; he established good relationships, he has credibility with them and with us as staff.*”

(Care-coordinator)

“*Having a job gives me a reason to live. When working, I can show ‘I am like you, I am like others’. Especially because of the stigma in our community. Work is a lifesaver. It’s not the money, it’s therapeutic. You need a structure, rather than days in front of the X-box, you need to get out. I’m in control, but* [Employment Specialist] *is there to support me if I need him*.”

(Client referred to Employment Specialist)

“*Dealing with your own ill-health is hard enough. Getting into work is another big battle. I went for jobs but got nowhere on my own through the universal job centre. On your own, you don’t know if you are coming or going. My condition means I can only take in so much, it’s the stress that gets you, lack of sleep. I need structure*.”

(Client referred to Employment Specialist)

“*I’ve got a job as a kitchen assistant. I didn’t want to be out in public. I would never have considered something like that—me in a kitchen? Never worked in one before, thought I might spill everything. XXX said try it, that working with food can be therapeutic. I’d never thought of that. He gave me the confidence to apply. It’s perfect hours and fits with the kids and it’s working out really well.*”

(Client referred to Employment Specialist)

“*They gave me a leaflet, and I read it. You need to realise that I need written information in order to absorb facts. The leaflet was the most important thing that sold it. There were bullet points, it was practical, it said the service can help with this and this. It was in black and white in simple English. And it was in my pocket and gave me a boost. It led me to say to myself: ‘OK, now I can do things’*.”

(Client referred to Employment Specialist)

“*This service? It’s got to carry on!*”

(Client referred to Employment Specialist)

#### 3.3.2. Strengths of IPS Workers

Interviewees described the employment specialists, who worked as members of clinical teams in the sites, as kind and professional; providing a ‘person-centred’ service, being approachable, non-judgmental and able to build trusting and constructive relationships.

“*The Employment Specialist was great. Inspiring, passionate but reacts to emotions, flexible… To be good, he has to be up on mental health issues and I know he is—some people are really conservative… Has to have the right personality, because even if you don’t get a job, you can benefit from the process of having someone like* [the employment specialist]”

(Client referring to the employment specialist).

“…*trust is a big issue. A few months ago, my CPN thought I was ready to work, and I was told there was a person who could help. I didn’t believe it*.”

(Client referring to the employment specialist).

“[Employment specialist] *came to meet me five months ago, along with my care-coordinator, and I thought ‘I’m not sure about this’. I thought it would be the same experience… fruitless. But there was no pressure on me and nothing to lose*.”

(Client referring to the employment specialist)

“*When I met [Employment Specialist], it was chilled out, in a coffee shop. We slowly talked about the situation and met a week later. I could tell that he saw me as a person and would ask ‘are you ready?’*.”

(Client referring to the employment specialist)

“*He gave me some options. I needed digest it all. XXX helped me with filling out applications. A burden was lifted off my shoulders, I didn’t know even how to say ‘dear sir or madam’, it would have been Mission Impossible*.”

(Client referring to the employment specialist)

“*He said it was up to me whether to post it, as he knew I might change my mind, and didn’t want to pressure me. He even phoned me on the Monday morning before the interview and guided me, got me to keep it simple, got me to practice over and over again. This was important, it got me the job. He gave me bullet points, kept it simple, he understands how I take things in. If it wasn’t for* [Employment Specialist] *I would not have got it, I would have given up. He said be yourself*.”

(Client referring to the employment specialist)

“[Employment specialist] *knows if you’ve had a bad day. He is more of a friend than a professional. The service is better than the job centre, there’s more of a connection. [Employment specialist] understands me. He has a rapport, he creates trust and has empathy. He asks me how I feel and didn’t just urge me to apply for hundreds of jobs*”.

(Client referring to the employment specialist)

“…*like a great taxi driver. You don’t want to sit with a smelly taxi driver. I look forward to seeing him. He has helped me get up in the morning. He speaks the lingo and has cred, knows the jargon*…”

(Client referring to the employment specialist)

#### 3.3.3. The Value of Employment Specialists for Community Mental Health Teams

The IPS staff noted that they were positively received by their Community Mental Health Team (CMHT) colleagues. CMHT professionals reported having been aware of employment support as a gap in provision, prior to the IPS service, with demands for employment support coming from service users.

“*We try to explain things really well in initial meetings—dispel myths to the service users and seek to reassure them and provide a sense of hope. It’s all about building and maintaining relationships*.”

(Employment specialist)

“…*less anxiety about the model (after time), become a bit more flexible concerning attitudes to paperwork—the* [IPS] *‘review’ helped us to see flexibility inherent in model…Still need to educate care co-ordinations…get them into the discipline of seeing the benefits*…”

(Employment specialist)

CMHT professionals viewed the IPS approach as more meaningful than the previous ‘generic’ employment support. They welcomed the employment specialists who were seen as being able to lift the burden from busy care-coordinators who had neither the time nor expertise to support people into employment. One indicator of this positive response was the large number of referrals to the employment specialist early in the introduction of the new services. The CMHT team members had a willingness to refer and to undertake proactive identification of potential clients. The referrals made by staff were appropriate. Service users understood the nature of the service to which they were referred and were interested in seeking employment.

“*The team have done an incredible job in a short time. They’ve taken on board the model, which is the most important aspect…The targets seemed hard. I almost had a heart attack when I saw it. If we get over 40 I’d be pleased, I thought. 72 now! And this is incredible thinking of this area as a place of high unemployment and low growth. We’ve exceeded expectations—this has smashed where we were*…”

(Regional coordinator)

Interviewees across all sites emphasised the practical benefits of having employment specialists working alongside mental health teams. When an employment specialist worked across two teams, their visibility was undermined, and team members found them less easy to contact. Most people reported that employment specialists developing strong relationships with mental health teams is crucial to IPS services getting timely referrals of people wanting help with work, and to raising the profile of employment within clinical teams.

“*In a client I am looking for an explicit reference to wanting to work, or I know the signs intuitively, if there’s discussion on where they go from here and what helps, if I see they want to be self-sustaining, or want to be busy… also have conversations sometimes with the Employment Specialist before mentioning it to client… I can rely on those conversations about how client is doing, for example a health visitor reported something to me and I can ask* [Employment Specialist] *to keep an eye on them. [The Employment Specialist] is there for the long haul. The service is a really effective antidepressant…but this is more than about just minimising challenges—it transforms lives*”

(Health professional)

#### 3.3.4. Challenges to Implementation Fidelity

The ways in which the IPS teams were started up, their management and supervision, and learning and development emerged as clear themes.

Implementation managers and team leaders were responsible for developing systems and processes to enable IPS teams to operate effectively. They understood the importance of developing firm foundations from the start, whilst at the same time developing plans to sustain the service. Their early tasks included recruitment and induction of employment specialists; awareness raising among mental health teams and service users; instigating policies and procedures for the new team; implementation of referral processes; developing IT systems which allowed shared record keeping; and setting up monitoring processes. Several sites reported delays in recruiting new staff and noted the importance of developing good relationships with human resource departments.

Other early challenges that were reported included:

- Finding office space for the implementation manager and team supervisor
- Identifying resources to create IPS employment specialist posts
- Setting up internal referral systems from clinicians to employment specialists
- Dealing with separate health and social care systems
- Attempting to integrate or work in complementary ways with third sector employment support organisations.

The rapid influx of referrals, whilst indicating early success, led to immediate pressures on the new services and the sites managed this in different ways. For example, in Northamptonshire, the new employment specialist’s supervisor compiled a list of people that care co-coordinators were considering referring, and triaged them before the Employment Specialist began work, thus creating a caseload of about 16 clients for him to work with.

“*The team have done an incredible job in a short time. They’ve taken on board the model, which is the most important aspect…The targets seemed hard. I almost had a heart attack when I saw it. If we get over 40 I’d be pleased, I thought. 72 now! And this is incredible thinking of this area as a place of high unemployment, with low growth; We’ve exceeded expectations—this has smashed where we were*…”

(IPS coordinator)

It was important to clearly define the roles of the implementation managers and team leaders. Doing so helped in the success in setting up systems. The team leader’s focus was largely operational and the implementation manager’s largely strategic. Success for a team leader relies on management and supervision expertise alongside the experience of managing a caseload. 

The three wave-two sites, which began 8–9 months after the wave-one sites, benefited from the creation of systems and resources developed by the wave-one sites, such as information for referrers and service users, data monitoring processes and reporting proformas.

Clear management and supervision arrangements are vital for employment specialists to avoid confusion about accountability and priorities. On the whole, employment specialists were managed within CMHTs but were supervised by team leaders. Employment specialists valued the presence of a good supervisor and many employment specialists welcomed “having a foot in both camps” and receiving different sorts of support and advice. A few felt that the slight confusion over having “many masters” (as one employment specialist put it) was outweighed by the diversity of communication. They thought that supervision helped in their management of caseloads and this was seen as important to ensure that employment specialists are able to offer a high fidelity IPS service.

Learning and development were seen as crucial for the creation of a growing IPS workforce, and this included opportunities to share learning as well as formal training. The employment specialists and clinical staff reported that they benefited from working with people from a diverse range of professional backgrounds and specialisms (for example, in benefits advice or employer engagement). However, many interviewees noted that passion, commitment and person-centred values were as important as professional skills.

#### 3.3.5. Working across and Outside of the Mental Health Organisation

During the site visits, several organisational factors were identified that contributed to successful implementation and sustainability of the IPS services. Strategic oversight and corporate support for IPS were critical success factors and were evident in all sites. The implementation manager had an important role to play in developing strategy, making sure that senior leaders were aware of the work, valued it, received the right information at the right time and were able to link it to corporate priorities as well as the future of the health economy. It was important to align monitoring, reporting and performance management processes with mainstream programmes, executive teams and the board. Important in this process was the development of trusted and influential relationships. Some sites created strategic champions at executive and board levels. A further role of the implementation manager was to connect horizontally to work closely with the team leader to link the work with CMHTs and clinical management teams.

In the interviews, several trust leaders expressed enthusiasm for IPS, seeing it as providing a serious focus on employment support, after years of intermittent funding, fragmented vocational services and reliance on individual champions. Some liked its innovative element, particularly where a site prided itself on having a broad culture of innovation in mental health services. The existing presence of ‘pioneers’ within the organisation who had already used IPS was noted to be important as gaining access to wider, collective corporate support was often dependent on an early enthusiast at middle or senior level.

There was a genuine passion amongst leaders for the work (sometimes arising from personal experience): “once you have that emotional connection, the penny drops, and you see the need for a sustainable package that reduces reliance on services” (senior manager). Many senior level interviewees disclosed personal experiences of having been affected by mental health problems, or of having family members or friends affected. This ‘lived-experience’ aspect of delivering services seemed important in driving both individual workers and clinicians and more senior leaders.

Working in partnership with other local organisations was viewed as a key to success for many IPS services. Several teams developed relationships with the voluntary sector over time. In some sites, partnerships with the voluntary sector were built into early commissioning intentions for models that were recovery based and so would allow the voluntary and statutory sector to work together. At the time of the later site visits, all sites were sustaining their IPS services as part of a spectrum of ‘employment support’ and were keen to make sure partnerships worked well between statutory and voluntary services. For example, the Bradford site worked well with voluntary sector partners, despite initial difficulties and developed a more strategic and integrated approach to work between statutory and voluntary sector providers.

Several IPS teams found early ways to raise the visibility of the work via public meetings, securing visits to the team from chairs and senior managers, getting articles in the local media and winning trust team awards. The host trusts often ensured corporate visibility for the work through successful awareness raising sessions for board and executive teams. Getting Centre of Excellence status, for example in Bradford and Lincolnshire, was also seen as a great “marketing tool” (see: https://www.centreformentalhealth.org.uk/ips-centres-of-excellence).

#### 3.3.6. Sustaining the IPS Services

The sustaining and continued funding of the IPS services proved to be difficult and varied across the six sites (see Table 1). The demonstration of successful employment outcomes for the services provide no assurance of funding success.

“*Even if the project demonstrates great outcomes, there’s no guarantee the trust will be able to prioritise it*”

(Board Member)

In general, at the corporate level, there was support for the development of IPS services, but such warm words were not matched by giving priority to the successful IPS services. Table 1 shows that some sites were successful in maintaining their services. Northamptonshire secured continued funding. Bedfordshire maintained their funding but, in the neighbouring area of the same site, Luton’s services were not preserved. Berkshire also managed to hold on to their two new IPS workers.

Several factors appeared to influence the successful maintenance of the IPS services. First, placing the IPS within a broader area of strategy, broadly that of a ‘Recovery-oriented’ plan. Second, aligning decision making at the trust corporate level, commissioning level, and the CMHT/clinical management level.

Northamptonshire secured the commitment of the trust’s board to keep their funding. In Bedfordshire, the commissioners, local authority and health services worked together. The mental health commissioners ring-fenced funding for ‘recovery’, making it possible for the implementation manager to bring IPS into this recovery strategy. In Northamptonshire, local commissioners and the NHS Trust were committed to IPS. One driver for this was the National Guidance and Quality Standards for Psychosis Services, which recommend the use of supported employment in services for people with psychoses [20,21,22].

## 4. Discussion

It is widely acknowledged that the implementation of IPS services is a challenge, despite the extent of evidence for its efficacy [1,12]. The *Making IPS Work* initiative has highlighted some of the difficulties facing implementation, particularly those of sustaining funding and continuing the initiatives. Despite these difficulties, several areas of success were seen, and lessons were learned.

The process of implementation of innovations in mental health services is not well understood [23] and in the discussion of the present findings we have focused on the context, process and outcomes of the initiative, as recommended by Brooks et al. [23].

### 4.1. Context

The drivers for expanding the development of IPS in the UK include government policy to improve the employment of people with disabilities [24]; the social inclusion of people with mental health conditions, including their employment opportunities [10,25]; and the increasing international evidence for the efficacy of IPS [2]. However, the implementation of IPS in the UK has thought to have been hampered by the attitudes, expectations and organisation of mental health services [12]. In general, barriers to innovations in mental health services have been ascribed to corporate departments and middle management [23]. Nevertheless, this evaluation suggests that there is an increasing acceptance of the value of IPS across the organisations studied.

Across all sites there was little resistance to the setting up of the IPS services and they were positively regarded by providers and users of the services. For the people involved in *Making IPS Work* the benefits went beyond the satisfaction of individuals who achieved their aim of entering paid employment. Interviews with NHS staff, senior managers, team leaders and existing employment workers revealed that they appreciated the dedicated resource of an implementation manager and team supervisor, with the Centre for Mental Health supporting them. This background support included training, supervision and access to centres of best practice in IPS, to ensure that the new sites were established with high fidelity to the IPS standards. The NHS managers and care coordinators were keen that service users should have access to supported employment, but also recognised that this required time and expertise not generally available among the community team professionals and support workers. In the interviews and group discussions people reported striking stories of success from IPS teams and health professionals for people with particularly challenging circumstances (for example, a criminal record, literacy issues or having lived in deprived areas). This positive view is shared by clients, employment specialists, health professionals, managers and senior leaders. This was viewed as having a knock-on effect on staff, boosting morale and professional confidence within teams.

### 4.2. Process

Several themes emerged from the implementation project. It is important to get systems in place at an early stage. For example, recruitment procedures should be rigorous and timely: Management and supervision arrangements should be made clear; and referral procedures should be kept as simple as possible. Clear goals need to be identified early and robust reporting, monitoring and performance management systems should be set up.

IPS can be considered as a disruptive innovation, thus it is important to focus on the management of change. This means recognising that many aspects of implementation rely on ‘informal’ approaches and building trusting relationships between key stakeholders. There is a need to focus on communication strategies at different levels of the organisation. The implementation managers and team leaders should be in a position to provide influential, supportive and collaborative leadership. Creating the climate for change to occur may mean taking an organisational development approach to implementation: Recognizing the strategic element of implementation at an early stage and identifying additional IPS champions, allies, decision makers and budget holders. It is valuable to ensure executive level accountability, responsibility and influence. Commissioners of services, executive and senior managers and IPS champions need to understand the scale of the change and communicate this to others.

The IPS services in this project were embedded in mental health teams and had varied success in engaging with external stakeholders, such as local voluntary sector organisations, employers and the Department for Work and Pensions (DWP). Conducting a local stakeholder mapping and communication plan can be beneficial in improving engagement. Engagement with the voluntary sector may be hampered by a fear that IPS services may threaten the vocational services traditionally delivered by the voluntary sector. Several sites addressed this by viewing IPS as one element in a spectrum of employment support, from voluntary work, through training to paid employment. For employers, it may be valuable to make the link as early as possible between IPS for individuals and a more corporate and strategic approach (awareness raising in the community; tackling stigma; support in the workplace) and ensuring that employment specialists are confident in engagement with employers.

### 4.3. Outcomes

The *Making IPS Work* initiative was concerned with achieving two main outcomes: The placement of people into open employment and the sustaining of the IPS services themselves. The former outcome was successfully achieved across all sites, but the latter outcomes were not always achieved.

IPS is one of many interventions and approaches that are considered by large health and social organisations which may face budgetary and other pressures when assessing their priorities. Standing on its own, IPS is likely to get drowned out for consideration by the other competitors. It was striking that one of the successful sites placed IPS as an element in the broader strategic objective of improving the trusts recovery-orientated services. This meant promoting IPS as part of a trust-wide commitment to a more ‘psychosocial’ approach to care. Taking this approach is consistent with the recommendations of another nationally funded project, the *Implementing Recovery through Organisational Change* (ImROC) project, which recommended that supported employment be an integral part of the development of recovery-orientated mental health services in the UK [26]. Many NHS Mental Health trusts in the UK have developed recovery-orientated strategies and recovery-orientated mental health services and similar person-centred approaches are advocated in national policies in England [27]. IPS is also recommended by NICE as part of quality-based services for people with psychoses [20,21,22].

A further threat to the development of IPS services across the six sites was the wider influences of the reduction in funding to NHS bodies and national budgetary constraints. This provides the context that will have contributed to the lack of maintenance of the IPS services in the sites and emphasises the need for agreement across health trusts and commissioners to fund develop supported employment services as part of their strategic plans.

### 4.4. Limitations of the Evaluation

There are several limitations. Only two of the five sites had fidelity reviews and it thus remains uncertain as to whether all sites where operating at a good level of fidelity. Nevertheless, all sites attained the targets for their employment outcomes. We did not collect sufficient detail on the employment outcomes, types of jobs and the views employers, thus limiting the knowledge about the quality of the jobs obtained.

## 5. Conclusions

Not only is there considerable evidence for the efficacy of IPS, there is also a call for the implementation of IPS from people who experience mental health conditions, clinicians, health and social services and national policy. Every area in England would like to see an IPS service within secondary mental health care. On the whole, NHS trusts, social care and third sector support employment providers are keen to participate in the delivery of IPS. Setting up a local IPS service with successful employment outcomes is readily achievable and valued by stakeholders. Nevertheless, the growth of these services and their maintenance are difficult to achieve when set against the ongoing cost pressures on the NHS, caused by the increase in demand on secondary services and the costs of maintaining quality. Embedding supported employment within a wider strategy of improving psychosocial approaches to care and recovery agreed at a local level between providers and commissioners of services may offer a means of increasing the number of IPS teams and sustaining them over longer periods.

## Figures and Tables

**Table 1:** The sites and Funding for Employment Specialists.

Site	Start Date	Internal Resources	External Resources	Sustainability
Berkshire	28 January 2015	2 IPS posts in Reading and Slough CMHTs.	None achieved	The 2 new posts continued after the grant period.
Lincolnshire	28 January 2015	Conversion of 2 existing supported employment posts to IPS. Team Leader post fulfilled by part-time senior occupational therapist.	None achieved	The 2 posts continued after the grant period, but the senior OT post disappeared when the postholder left.
Bradford	28 January 2015	Four posts created with internal investment of surplus and one post funded by salary released from seconded staff to grant-funded team leader post.	None achieved	The five posts and team leader continued after the grant period, but recurrent revenue funding was under threat and required matched disinvestment to become fully sustainable.
Wiltshire	1 October 2015	None required	The IPS team was fully funded by Wiltshire Council and Wiltshire Clinical Commissioning Group (CCG)	Wiltshire joint commissioning provided funding for a team leader and 5.5 employment specialists with a 3–5-year contract.
Bedfordshire and Luton	28 September 2015	Four posts in Luton created with internal resources (primarily the salaries released from the secondment of two members of staff to grant funded posts). Three posts in Bedfordshire already in existence.	None achieved	The Bedfordshire posts continued but as commissioners were unable to pick up funding for the Luton posts these were made redundant 6 months after the grant period ended.
Northamptonshire	1 December 2015	Salary of seconded staff member funded a post in the forensic service. An existing employment post within the Early Intervention Service provided a second IPS post.	The secondment of a post funded by an outside agency was added to the IPS team during the grant period	Although the seconded post ended, the trust continued to fund the team leader in addition to the forensic service post and early intervention post.

**Table 2:** Interviews undertaken across the sites.

Trust	Visit 1		Visit 2		Visit 3		Number of Interviews Undertaken
	Staff	Service Users	Staff	Service Users	Staff	Service Users	
Berkshire	15	0	18	7	16	4	49 staff 11 service users
Lincolnshire	9	5	9	4	11	7	22 staff 16 service users
Bradford	12	0	17	3	13	3	42 staff 6 service users
Northamptonshire	9	2	13	6	11	6	33 staff 14 service users
Wiltshire	17	6	15	2	12	6	44 staff 14 service users
Luton and Bedfordshire	16	0	14	4	15	0	45 staff 4 service users

**Table 3:** Referrals and work outcomes in each site.

Site	Referrals and Jobs Achieved
Berkshire	240 referrals and 74 jobs achieved—31% success
Bradford	193 referrals and 78 jobs achieved—40%
Lincolnshire	170 referrals and 65 jobs achieved—38%
Luton & Bedfordshire	264 referrals and 114 jobs achieved—43%
Northamptonshire	139 referrals and 27 jobs achieved—19%
Wiltshire	155 referrals and 63 jobs achieved—41%

## Abbreviations

The following abbreviations are used in this manuscript:

IPS	Individual Placement and Support
NHS	National Health Service
CCG	Clinical Commissioning Group
OT	Occupational Therapist
CMHT	Community Mental Health Team
CV	Curriculum Vitae
DWP	Department for Work and Pensions
NICE	The National Institute for Health and Care Excellence
